# The Polyphenol Oleuropein Aglycone Protects TgCRND8 Mice against Aß Plaque Pathology

**DOI:** 10.1371/journal.pone.0071702

**Published:** 2013-08-08

**Authors:** Cristina Grossi, Stefania Rigacci, Stefano Ambrosini, Teresa Ed Dami, Ilaria Luccarini, Chiara Traini, Paola Failli, Andrea Berti, Fiorella Casamenti, Massimo Stefani

**Affiliations:** 1 Department of Neuroscience, Psychology, Drug Research and Child Health, Division of Pharmacology and Toxicology, University of Florence, Florence, Italy; 2 Department of Experimental and Clinical Biomedical Sciences, University of Florence, Florence, Italy; 3 Research Centre on the Molecular Basis of Neurodegeneration, University of Florence, Florence, Italy; Nathan Kline Institute and New York University Langone Medical Center, United States of America

## Abstract

The claimed beneficial effects of the Mediterranean diet include prevention of several age-related dysfunctions including neurodegenerative diseases and Alzheimer-like pathology. These effects have been related to the protection against cognitive decline associated with aging and disease by a number of polyphenols found in red wine and extra virgin olive oil. The double transgenic TgCRND8 mice (overexpressing the Swedish and Indiana mutations in the human amyloid precursor protein), aged 1.5 and 4, and age-matched wild type control mice were used to examine in vivo the effects of 8 weeks dietary supplementation of oleuropein aglycone (50 mg/kg of diet), the main polyphenol found in extra virgin olive oil. We report here that dietary supplementation of oleuropein aglycone strongly improves the cognitive performance of young/middle-aged TgCRND8 mice, a model of amyloid-ß deposition, respect to age-matched littermates with un-supplemented diet. Immunofluorescence analysis of cerebral tissue in oleuropein aglycone-fed transgenic mice showed remarkably reduced ß-amyloid levels and plaque deposits, which appeared less compact and “fluffy”; moreover, microglia migration to the plaques for phagocytosis and a remarkable reduction of the astrocyte reaction were evident. Finally, oleuropein aglycone-fed mice brain displayed an astonishingly intense autophagic reaction, as shown by the increase of autophagic markers expression and of lysosomal activity. Data obtained with cultured cells confirmed the latter evidence, suggesting mTOR regulation by oleuropein aglycone. Our results support, and provide mechanistic insights into, the beneficial effects against Alzheimer-associated neurodegeneration of a polyphenol enriched in the extra virgin olive oil, a major component of the Mediterranean diet.

## Introduction

Alzheimer’s disease (AD) is the most common form of dementia affecting a large proportion of aged people in the developed countries, where it represents a severe burden for its dramatic social impact and for national health budgets. The key histopathological sign of AD is the presence, in several brain areas, of intracellular neurofibrillary tangles of hyperphosphorylated tau, of minute extracellular amyloid deposits found in diffuse and senile plaques and around cerebral vessels and of dystrophic and degenerating neurites [1], [2]. Presently, functional alterations and behavioral deficits that characterize AD are thought to result primarily from the presence of plaque deposits [3], whose main component is a polymeric fibrillar form of the 42 amino acid peptide (Aβ42) generated by proteolysis of the membrane amyloid precursor protein (APP) [3]. Plaque load results from complex equilibria between Aβ deposition and clearance, where autophagy, a lysosome-mediated catabolic pathway responsible for turnover of long-lived proteins and organelles, appears to perform a key role [4], [5]. Autophagy protects neurons against Aβ-induced cytotoxicity suggesting its possible role in Aβ clearance [6]; moreover, the induction of autophagy by rapamycin in mouse models of AD results in a decreased accumulation of Aβ and aggregated tau [7].

More recently, the interest in deciphering the relation between plaque burden, tissue functional impairment and neuronal death has focused the importance, as the main toxic species to neurons, of the oligomeric pre-fibrillar assemblies originating at the onset of fibril growth [8]–[12]. Accordingly, the research of treatments able to delay AD occurrence and to relieve its symptoms has shifted from the development of molecules interfering with fibril growth to that of molecules able to counteract the appearance of toxic oligomeric intermediates.

Focusing dietary regimens associated with a reduced risk of AD in the aged population can be useful to find molecules exploitable for AD prevention and therapy. Mounting evidence supports the beneficial effects of the Mediterranean diet (MD) in preventing age-related dysfunctions, cancer, neurodegenerative diseases and in attenuating AD-like pathology and cognitive deterioration [13]–[18]. In particular, MD appears to be effective against mild cognitive impairment and its conversion to AD [13]. Studies in rodents suggest that diet supplementation with polyphenol-rich components of the MD such as red wine and extra virgin olive oil (EVOO) improves learning and behavioral deficits associated with aging and disease [19], [20]. In addition, several reports, including the “Three city study” [21] support a strict association between many protective effects of the MD and the sustained assumption of EVOO. In particular, a number of polyphenols and secoiridoids found in EVOO, including oleocanthal, hydroxythyrosol and oleuropein aglycone (OLE), have been considered potentially responsible for the beneficial effect of MD [22]–[26]. Here we describe a comprehensive study on OLE protection against AD in the TgCRND8 (Tg) mouse model of Aβ deposition. Our behavioral, biochemical and histochemical data in OLE-fed animals agree with the key role of amyloid plaques as the main responsible of neuronal network impairment in brain [27]; they also support the beneficial effects of dietary EVOO and highlight the possibility that OLE intake may be useful to prevent AD or, at least, to delay the appearance, and to reduce the severity, of its symptoms.

## Materials and Methods

### Ethics Statement

Transgenic CRND8 mice encoding a double-mutant of APP695 [28] and wild type (wt) control littermates were used following the ECC (DL 116/92, Directive 86/609/EEC) and National guidelines for animal care. The protocol was approved by the Committee on the Ethics of Animal Experiments of the Italian Ministry of Health (Permit Number: 283/2012-B).

### Animals

The following 2 age groups of mice (equally divided for sex) were used: 1.5 month-old wt (n = 16) and pre-plaque Tg (n = 16) mice, 4 month-old wt (n = 16) and Tg (n = 16) mice. Different diet treatments were equally given to each age group of wt and Tg mice: 8 weeks with a 5.0% fat diet (10 g/day per mouse) either alone (untreated) or containing OLE (50 mg/kg of diet) (OLE-fed). We used a modified low-fat AIN-76A diet composed of 50.0% sucrose, 5.0% fat, 20.0% casein, 15.0% corn starch, 5.0% powdered cellulose, 3.5% AIN-76 mineral mix, 1.0% AIN-76A vitamin mix, 0.3% DL-methionine and 0.2% choline bitartrate (Piccioni, Italy).

### Oleuropein Deglycosylation

Oleuropein (Extrasynthase) deglycosilation was performed according to Konno et al. [29] with minor modifications [23]. Briefly, a 10 mM solution of oleuropein in 0.1 M sodium phosphate buffer, pH 7.0, was incubated with 28.7 I.U./mL of β-glycosidase overnight at room temperature in the dark. The reaction mixture was centrifuged at 10,000×g for 15 min to precipitate the aglycone. The complete oleuropein deglycosylation was confirmed by assaying the glucose released in the supernatant with the Glucose (HK) Assay Kit (Sigma). GC-MS analysis showed the absence of any oleuropein in the precipitate and the substantially total recover of OLE in the same precipitate. A 100 mM OLE stock solution in DMSO was stored protected from light and diluted in oil immediately before diet supplementation.

### Behavioral Experiments

At the end of the diet treatment, 3.5 month-old and 6 month-old OLE-fed and untreated Tg and wt mice were pooled according to the treatment and genotype and behaviourally tested. The apparatus and procedures used for the “Step-Down” inhibitory avoidance test were previously described [30]. The apparatus was an open field plexiglas box (40×40 cm) with a steel rod floor and a plexiglas platform (4×4×4 cm) set in the centre of the grid floor to which intermittent electric shocks (20 mA, 50 Hz) were delivered. On day 1 (training test, TT), each mouse was placed on the platform and received an electric shock for 3 s when it stepped down placing all paws on the grid floor. Responsiveness to the punishment in the TT was assessed by animal vocalization; only those mice that vocalized touching the grid (about 70% of mice) were used for retention test (RT). 24 h after TT, each mouse was placed on the platform again (RT). The latencies were measured, considering 180 s as the upper cut-off, during TT and RT. The tests were carried out between 10∶00 A.M. and 1∶00 P.M.

For object recognition test (ORT), we modified our previously described method [31] using a white box (60×50×25 cm) with a grid floor covered by white filter paper. A 75 Watt lamp was suspended 50 cm above the box. Mouse behaviour was recorded by a video-tracking/computer-digitizing system (HVS Image, UK). The day before testing, the mice were allowed to explore the box for 5 min. A session of two trials (T1 and T2) at 60 min interval was given on the test day. In T1, the time spent by each mouse exploring two identical 8.0 cm side grey cubes presented for 10 min in two opposite corners of the box was recorded. During T2, one of the cubes was replaced by a 8.0 cm side grey cylinder, and the mice were left in the box for 5 min. The time spent for the exploration of the familiar (F) and the new (N) object were recorded and a discrimination score (D = N/N+F) was calculated. A discrimination score above 0.5 indicates the ability of mice to discriminate between the familiar and novel objects while a score below or equal to 0.5, reflecting a novel object exploration time less or equal to the half of the total time spent between the two objects, indicates memory impairment in this task [32], [33].

### Animal Tissue Processing

After completing the behavioral tests, the mice were sacrificed by cervical dislocation and the brains were rapidly removed and divided sagittally. For protein analysis, cortical and hippocampal samples from one hemibrain were immediately sectioned, snap-frozen and stored at −80°C. The other hemibrain was postfixed in phosphate-buffered 4.0% paraformaldehyde, pH 7.4, at 4°C for 48 h, rinsed in PBS and paraffin embedded for immunohistochemistry and Thioflavin S staining.

### Cell Treatment

N2a murine neuroblastoma cells (ECACC) were plated in MEM supplemented with non-essential amino acids, 10% FCS, antibiotics and glutamine, cultured 24 h, treated with different concentrations of OLE for increasing time periods and evaluated for viability by the NR Uptake assay, as previously described [24]. The autophagy markers were determined by western blotting on cell lysates.

### Immunohistochemistry, Histochemistry and Western Blotting

Histochemical and immunohistochemical analyses were performed on 5.0 µm coronal paraffin-embedded sections, as previously described [34]. The sections were incubated overnight at 4°C with the primary antibodies (Abs) (Table 1) diluted in 0.1 M PBS, pH 7.4, with Triton X-100 (0.3%) and BSA (5.0 mg/ml). For Aβ plaque identification and astrocyte staining with GFAP, tissue sections were incubated for 1.0 h with the biotinylated secondary Ab (Vector Laboratories, USA) diluted 1∶1000 in PBS 0.1 M/BSA (1.0 mg/ml); immunostaining was visualized using the avidin-biotin system (Vectastain) and 3,3′- diaminobenzidine plus Nickel (DAB Kit) (Vector Laboratories, USA) as the chromogen. Fluorescent immunohistochemistry experiments were as previously reported [35]. The sections were incubated for 1.0 h with blocking solution (BS) containing 0.25% Triton X-100, 5.0 g/l BSA and 5.0% normal goat serum for polyclonal Abs or 5.0% normal horse serum for monoclonal Abs in 0.1 M PBS, pH 7.4, and then overnight at 4°C with the primary Ab (Table 1). On day 2, the sections were incubated for 1.0 h in the dark with the appropriate fluorescent secondary Ab (Alexa Fluor 594- or 488-conjugated monoclonal anti-mouse and polyclonal anti-rabbit Ab, Invitrogen, USA) diluted 1∶400 in BS. For double immunostaining, the sections were incubated with the second primary Ab in the dark overnight at 4°C. On day 3, the slices were incubated in the dark for 1.0 h at room temperature with the second fluorescent Ab diluted 1∶400 in BS. Analysis of negative controls (not treated with the primary Ab) was simultaneously performed to exclude the presence of non-specific immunofluorescence staining, cross-immunostaining, or fluorescence bleed-through. Thioflavin S staining was as previously described [35]. The rehydrated sections were incubated with 0.25% KMnO_4_ for 4.0 min, washed with water, incubated with a 0.1% NaBH_4_ solution for 5.0 min and placed in phosphate buffer (411 mM NaCl, 8.1 mM KCl, 30 mM Na_2_HPO_4_, 5.2 mM KH_2_PO_4_), pH 7.2 for 30 min at 4°C. The sections were washed, incubated in 0.05% Thioflavin S (in 50% ethanol) for 8.0 min in the dark, washed twice in 80% ethanol for 10 s and incubated in phosphate buffer for 30 min at 4°C. The sections were cover slipped using Vectashield mounting medium with or without DAPI (Vector Laboratories, USA). Representative images were acquired by an Olympus BX63 microscope coupled to *CellSens Dimension* Imaging Software (Olympus, Italy). For western blotting analysis, tissue samples were homogenized in ice-cold RIPA lysis buffer, the cells were directly lysed in Laemmli sample buffer, and 40 µg of proteins were applied to SDS-PAGE (10% or 15% acrylamide resolving gel) [36] for electrophoresis. The separated proteins were transferred onto 0.45 µm nitrocellulose/PVDF membrane (Hybond-C, Amersham Life Science) and incubated overnight at 4°C with the primary Ab (see Table 1). The day after, the blots were incubated for 1.0 h with the HRP-conjugated secondary Ab (Biorad) diluted 1∶7500 in BS. The immunocomplexes were visualized using enhanced chemiluminescence (ECL, Pierce, USA) and acquired using ImageQuant 350 system (GE Healthcare, UK). Band densitometric analysis was performed using Image Quant TL software (GE Healthcare, UK). The bands were normalized to β-actin or GAPDH level. All primary Ab concentrations were titrated to provide optimal staining. The groups of untreated or OLE-fed wt mice were pooled for western blotting analyses.

**Table 1:** Antibodies employed in the study.

Antibody	Specific	Dilution		Host	Source
		WB	IHC		
**Aβ42**	Aβ peptide, aa 1–42	ND	1∶200	Rabbit	Millipore
**Cathepsin B**	Procathepsin B and mature Cathepsin B	1∶1000	1∶100	Rabbit	Millipore
**SQSTM1/p62**	SQSTM1/p62 protein	1∶1000	1∶200	Mouse	abcam
**Beclin 1**	Beclin 1 protein	1∶1000	1∶200	Rabbit	abcam
**LC3**	microtubule-associated protein light chain 3	1∶1000	1∶200	Rabbit	Novus Biologicals
**GFAP**	Glial fibrillary acidic protein	ND	1∶500	Rabbit	Dako
**Iba 1**	Ionized calcium binding adaptor molecule 1	ND	1∶250	Rabbit	Wako
**OC**	Amyloid fibrils and fibrillar oligomers	ND	1∶1000	Rabbit	CG Glabe
**P-p70S6K**	P-p70 S6 kinase (Thr389)	1∶1000	ND	Rabbit	CST
**p70S6K**	p70 S6 kinase	1∶1000	ND	Rabbit	CST
**GAPDH**	Glyceraldehyde 3-phosphate dehydrogenase	1∶1000	ND	Rabbit	Santa Cruz
**β-Actin**	C-terminal β-actin fragment (C11)	1∶5000	ND	Rabbit	Sigma-Aldrich

Legend: aa, amino acid; WB, western blot; IHC, immunohistochemistry, ND, not done.

### Determination of Aβ Plaque-load

To quantify Aβ plaque burden, cortices and hippocampi of the sections stained with an anti-Aβ42 Ab were digitized and acquired with an Olympus BX63 microscope equipped with *CellSens Dimension* software (Olympus, Germany) as previously reported [37]. 6 coronal brain sections, separated by 60 µm interval, from each mouse (4–5 animals/group) were analyzed. Plaque number and total area were determined automatically. Brain regions were based on the mouse brain atlas [38].

### ELISA

Soluble and insoluble Aβ fractions were isolated from cortex homogenates using a four step extraction protocol [39]. At each step, sonication in an appropriate buffer was followed by centrifugation at 100,000×*g* for 1 hr at 4°C. The supernatant was then removed, and the pellet was sonicated in the next solution used in the sequential extraction process. For four-step extraction, sonication of the frozen tissue began in Tris-buffered saline (TBS) (20 mM Tris and 137 mM NaCl, pH 7.6), which contained protease inhibitors (protease inhibitor cocktail from Sigma St. Louis USA). The next three sequential extraction steps used 1% Triton X-100 in TBS with protease inhibitors, 2% SDS in water with the same protease inhibitors, and 70% formic acid (FA) in water. SDS-soluble fractions were loaded directly onto ELISA plates, whereas FA-soluble fractions were diluted 1∶20 in a neutralization buffer (1 mol/L Tris base, 0.5 mol/L NaH_2_PO4) before loading. Levels of Aß40 and Aß42 were analyzed using an ELISA kit (Biosource, Camarillo, CA, USA) according to the manufacturer’s instructions. The plates were read at 450 nm using a plate reader (Molecular Dynamics, Sunnyvale, CA). All values were calculated as pmol per g based on the wet weight of brain cortical tissues.

### Thiobarbituric Acid Reactive Substances (TBARS) Assay

The mouse cerebral cortex was homogenized in RIPA buffer (10% w/v) containing the chelating agent diethylenetriamine pentaacetic acid (500 µM); 0.2 mL of suspension were added to 4.0 mL of 36 mM TBA solubilised in 10% CH_3_COOH, pH 4.0 [40]. After heating for 60 min at 100°C, the reaction was stopped by cooling the test tubes in ice for at least 2 h. 1.5 ml of n-butanol was added and each test tube was vigorously mixed for 30 s and centrifuged at 250×g for 10 min. The organic phase was separated and sample absorbance at 532 nm was determined spectrophotometrically. TBARS were quantified using 1,1,3,3-tetramethoxypropane as a standard. Each sample was evaluated twice.

### Data Analysis

One-way ANOVA, followed by Bonferroni's post-hoc test was used to analyse Beclin 1, LC3, Cathepsin B, p62 and Aß40 and Aß42 ELISA, TBARS levels, ORT and step down data. Unpaired two tailed Student’s t-test was used to analyse Aβ plaque burden. Statistical analyses were carried out with OriginPro 8.1 and statistical significance was defined as *P*<0.05. Data are reported as mean values ± standard error of the mean (S.E.M).

## Results

### OLE Improves Memory Deficits in TgCRND8 Mice

TgCRND8 mice are cognitively impaired since the age of 3 months [28], [30] and, as already reported by us [34], no differences are present between 3- and 7-month-old TgCRND8 mice. Therefore, in both genotypes mice of 3.5 and 6 months of age were grouped to increase the number of animals evaluated for cognitive function after 8 weeks of administration of food supplemented or not with OLE (n = 12/group for all groups). OLE treatment was well tolerated and no treated animals died.

**Figure 1:**
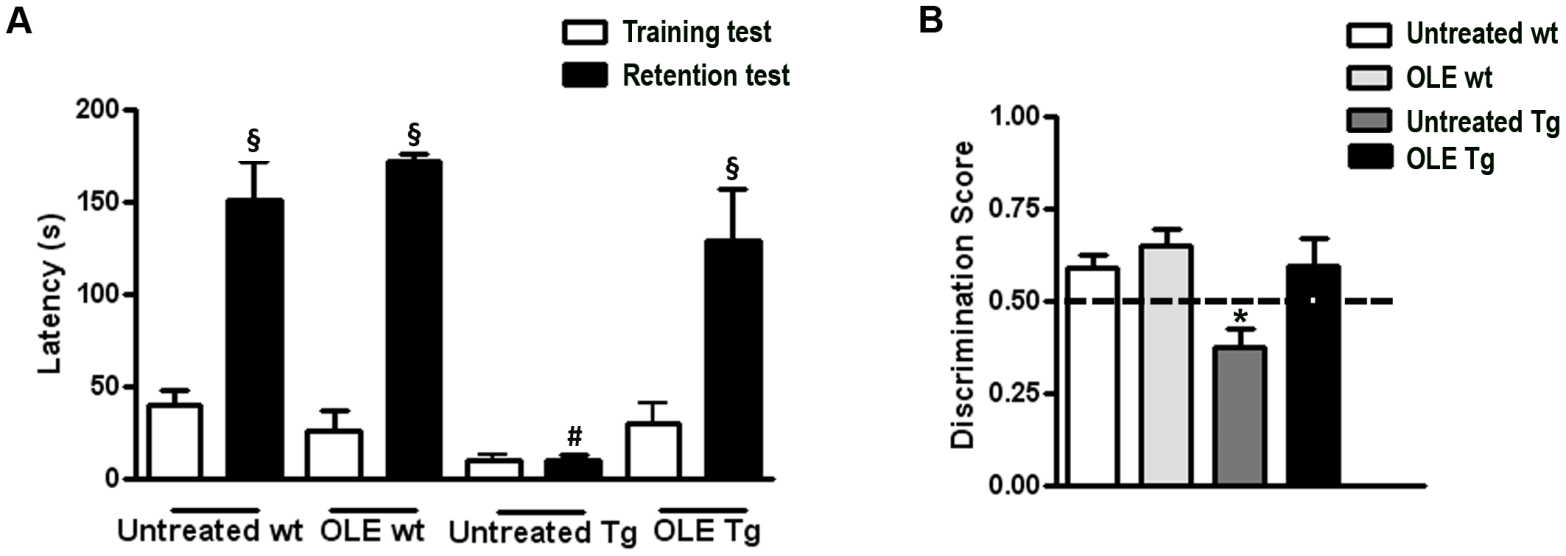
OLE restores cognitive performance in 3.5–6 month-old TgCRND8 mice. (A) Step down test: one-way ANOVA plus Bonferroni’s post comparison test shows a statistically significant increase in the mean retention latencies in untreated and OLE-treated wt and in OLE-treated Tg mice, as compared to their respective training latencies (§P<0.001). Untreated Tg mice do not show significant differences between training and retention latencies (P>0.05). The retention latencies of untreated Tg mice significantly differ from the retention latencies of all the other groups (#P<0.001). (B) ORT: in the T2 trial the discrimination index of untreated Tg mice significantly differ from the discrimination index of all other groups (*P<0.05). The dotted line indicates the chance level performance; Data are reported as mean values ± S.E.M. Number of animals: n = 12/group.

Memory performance was investigated by two widely used tests, the step down inhibitory avoidance and the ORT. No significant differences were observed between wt and OLE-fed or untreated Tg mice during the TT of the step down test. However, in the 24 h RT step-down latencies recorded for Tg mice were significantly reduced respect to wt mice and not significantly different from training latency (*P*>0.05), indicating that Tg mice were unable to memorize the punishment and to perform the inhibitory avoidance. OLE administration to Tg mice significantly improved their performance, that reached the level displayed by wt mice (Fig. 1A). These findings did not result from any difference in motility or exploratory activity, as evaluated in the rotarod and hole board tests (data not shown) nor from different sensitivity to pain in the investigated mice. Then, the same mice were tested for ORT with a retention interval of 60 min. In the T1 trial the exploration time of the familiar object was comparable in the four groups, where OLE-fed and untreated animals showed no deficiencies in exploratory activity, directional movement towards the objects and locomotor activity. In the T2 trial, untreated Tg mice exhibited impairments in novel object preference compared to wt mice, as shown by the significant reduction in the discrimination score (untreated Tg: 0.3767±0.051; untreated wt: 0.5909±0.037), (Fig. 1B). The ability of OLE-fed Tg mice to discriminate between the familiar and novel object was significantly improved (0.60±0.073) respect to that of untreated Tg mice and undistinguishable from that of wt mice (OLE-fed: 0.6525±0.044; untreated: 0.5909±0.037). Altogether, the results of the memory performance tests indicate that in our mouse model cognitive impairment is completely prevented/rescued by OLE administration to young/middle-aged Tg mice.

### OLE Modifies Aβ Burden in TgCRND8 Mice

**Figure 2:**
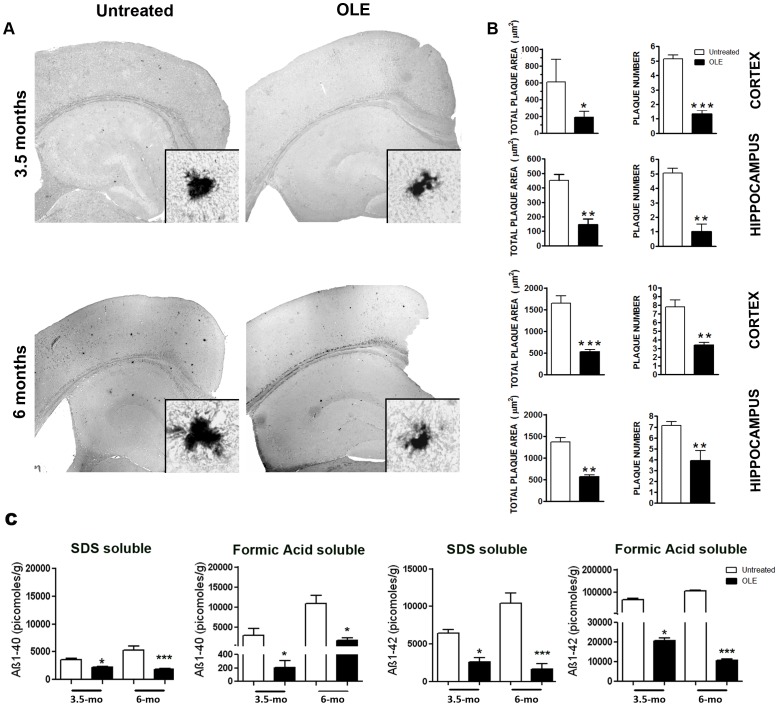
OLE reduces plaque burden in the cortex and hippocampus of TgCRND8 mice. (A) Representative photomicrographs of Aβ42 immunopositive deposits. (n = 5/group, six sections from each mouse). Insets: high magnification images of representative plaques. Scale bars = 500 µm applies to all reconstructed images and 20 µm to all magnified images. (B) Quantitative analysis of total plaque area and plaque number in untreated and OLE-fed Tg mice (n = 6 for 3.5 and 6 months Tg mice). (C) ELISA: cortical levels of SDS- and formic acid-soluble Aβ40 and Aβ42 peptides in OLE-fed and untreated Tg mice. Both Aβ40 and Aβ42 levels were significantly decreased in OLE-fed Tg versus age-matched untreated Tg (n* = *5/group) mice. *P<0.05, **P<0.01 and ***P<0.001. Data are reported as mean values ± S.E.M. mo = month-old.

Next, we checked whether the improved cognitive performance of OLE-fed young/middle-aged Tg mice resulted from any altered amyloid load respect to untreated Tg mice. Few, round-shaped, small to medium size plaques were detected by an anti-Aβ42 Ab in the cortex and hippocampus of untreated 3.5-month-old Tg mice (Fig. 2A). In 6-month-old Tg mice the Aβ load became heavier with a calculated total plaque area in the cortex and hippocampus of about 1600 µm^2^ and 1400 µm^2^, respectively (Fig. 2A), which was markedly reduced in the brains of age-matched Tg mice fed with OLE (Fig. 2A). Quantitative analysis of total Aβ plaque area and number in the cortex and the hippocampus revealed that the effect of OLE treatment was significant both in 3.5- and 6-month-old Tg mice (Fig. 2B), supporting a remarkable protective effect of OLE against early and middle stage of Aβ deposition. Aβ40 and Aß42 SDS and FA soluble fractions measured in the cortex of OLE-fed Tg mice of both ages were significantly reduced as compared to those measured in age-matched untreated Tg mice (Fig. 2C).

**Figure 3:**
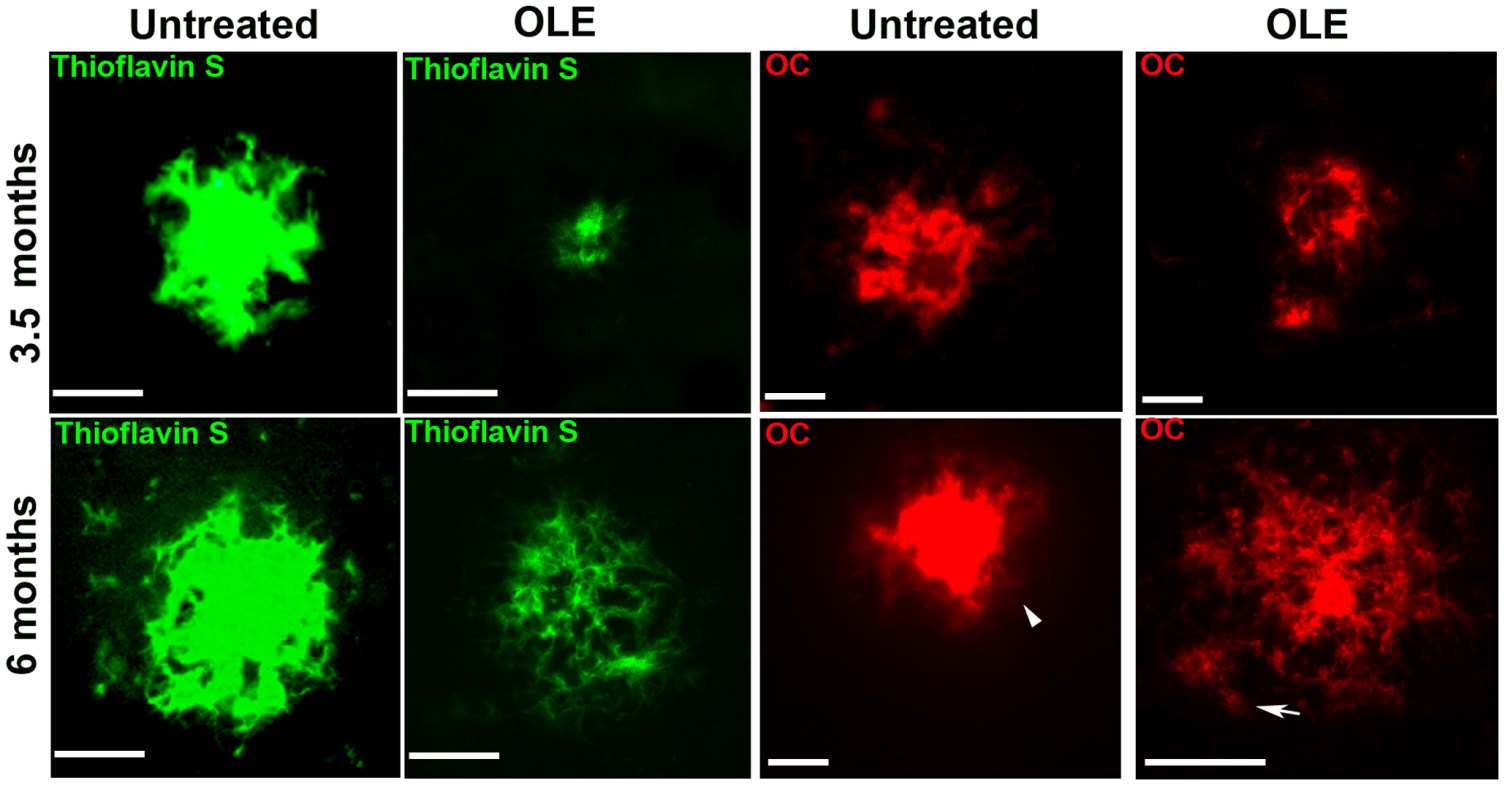
OLE modifies Aβ plaque load and morphology in the brains of TgCRND8 mice. Representative photomicrographs of Thioflavin S histochemistry (green) (n = 4/group) and OC immunolabeling (red) (n = 5/group) of amyloid plaques in the cortex of untreated and OLE-fed Tg mice. In the OLE-fed Tg mice of 6 months of age several radiating plaques with ribbon-like/diffuse core and fluffy deposits (arrow) are present. Arrowhead indicates dense core amyloid plaques. Scale bars = 25 µm.

Thioflavin S and the OC Ab which specifically recognizes fibrillar oligomers and amyloid fibrils [41], showed that in both the cortex (Fig. 3) and hippocampus of OLE-fed Tg mice of both ages the radiating plaques displayed a less dense core surrounded by fewer and smaller round-shaped deposits than in untreated Tg mice. Moreover, in the brain of 6-month-old Tg mice, displaying an intermediate stage of plaque deposition, feeding with OLE resulted in the presence of several radiating plaques with ribbon-like/diffuse core and in a remarkable presence of fluffy deposits (arrow in Fig. 3), whereas the plaques found in untreated Tg mice typically displayed a very dense core (arrowhead in Fig. 3). Altogether, these findings suggest that OLE, besides interfering with *de novo* amyloid deposition, favours preformed plaque disassembly.

### OLE Induces Autophagy in the TgCRND8 Brain

**Figure 4:**
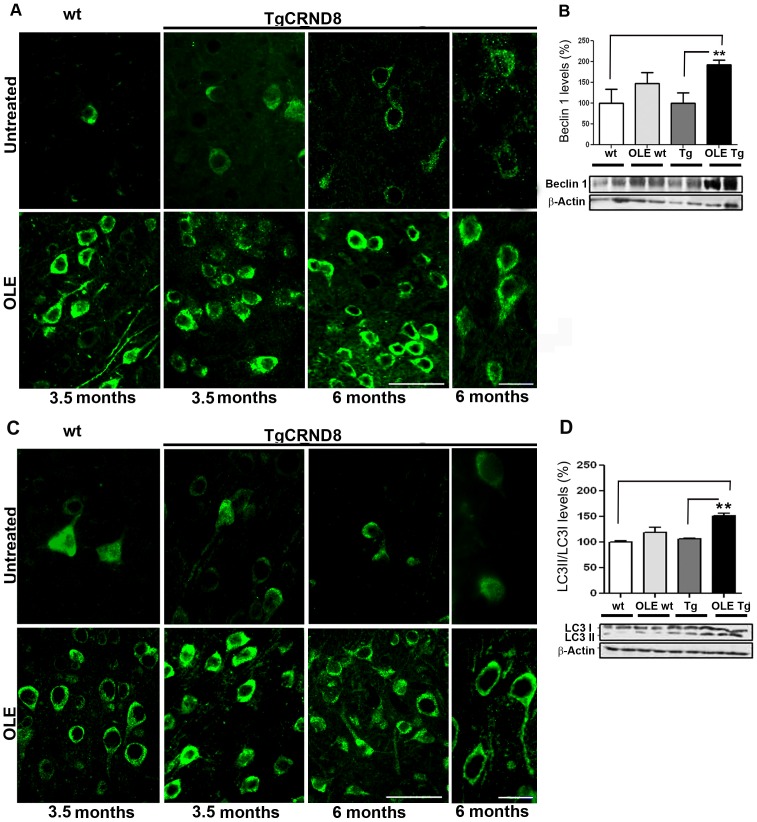
OLE increases Beclin 1 and LC3 in the cortex of wt and TgCRND8 mice. Representative images of Beclin 1 (A) and LC3 (C) immunoreactivity showing an intense bright and punctate Beclin 1 staining in the soma, perikarya and dendrites of neurons and strong and bright LC3 puncta in the neuronal cell bodies and processes of neurons in the somatosensory/parietal cortex of Tg mice and, to a lesser extent, in the wt mice fed with OLE, as compared to age-matched untreated Tg and wt mice (n = 5/group). Scale bars = 50 µm applies for the low magnification images and 20 µm applies for the high magnification images of untreated and OLE-fed 6-month-old Tg mice. (B) and (D) Western blotting analysis of Beclin 1 (B) and LC3 (D) levels in cortical tissue, exemplified for mice of 3.5 months of age, normalized for β-actin, (n = 6–7/group). LC3 levels are expressed as LC3-II/LC3-I levels. In the cortex of OLE-fed animals Beclin 1 levels show a trend towards an increase in the wt mice and in the OLE-fed Tg mice Beclin 1 and LC3 levels were significantly increased respect to age-matched untreated wt and Tg mice. (**P<0.01). Data are reported as mean values ± S.E.M.

It is widely recognised that autophagy protects neurons from Aβ-induced cytotoxicity, thus we investigated whether autophagy was involved in the interference of OLE with the de novo deposition and disassembly of Aß. Actually, an intense bright and punctate immunoreactivity for Beclin 1, a protein involved in the initiation and execution of autophagy, was detected in the soma, perikarya and dendrites of neurons in different layers of somatosensory/parietal (Fig. 4A) and entorhinal/piriform cortices of Tg mice fed with OLE as compared to age-matched untreated Tg mice with no apparent age-related differences. Although to a lesser extent than in OLE-fed Tg mice, Beclin 1 immunoreactivity was stronger also in OLE-fed wt mice than in untreated animals, as exemplified for 3.5-month-old mice in Fig. 4A, suggesting a general effect of OLE as inducer of Beclin 1 expression. In the cortex of OLE-fed animals of both ages, Beclin 1 levels showed a trend towards an increase in wt mice and a significant increase in the Tg mice compared to the respective age-matched untreated animals, as exemplified in Fig. 4B for mice of 3.5 months of age. OLE-induced autophagy was further confirmed by immunohistochemical analysis of LC3-II, the membrane-associated lipidated LC3 form that appears in newly formed autophagosomes [42]. Stronger and brighter LC3 puncta were detected in the neuronal cell bodies and processes in the somatosensory/parietal (Fig. 4C) and entorhinal/piriform cortices of Tg mice fed with OLE, as compared to age-matched untreated Tg mice. As for Beclin 1, LC3 immunoreactivity was stronger also in all OLE-fed than in untreated (Fig. 4C) wt mice. As for Beclin 1, in the cortex of OLE-fed Tg and wt mice of both ages LC3-II levels showed a trend towards an increase in the wt mice and a significant increase in the Tg mice respect to age-matched untreated wt and Tg mice, as exemplified for 3.5-month-old mice (Fig. 4D). Overall, our data indicate that OLE triggers autophagy not only in Tg but also in wt mice suggesting a possible beneficial effect of this polyphenol also against age-related neurodegeneration such as that occurring in sporadic AD, with an effect similar, at the molecular level, to that elicited by caloric restriction [43]. In general in the hippocampus autophagy was less evident, some Beclin 1 and LC3 puncta were detected in the CA1, CA3 and dentate gyrus of OLE-fed Tg mice only.

**Figure 5:**
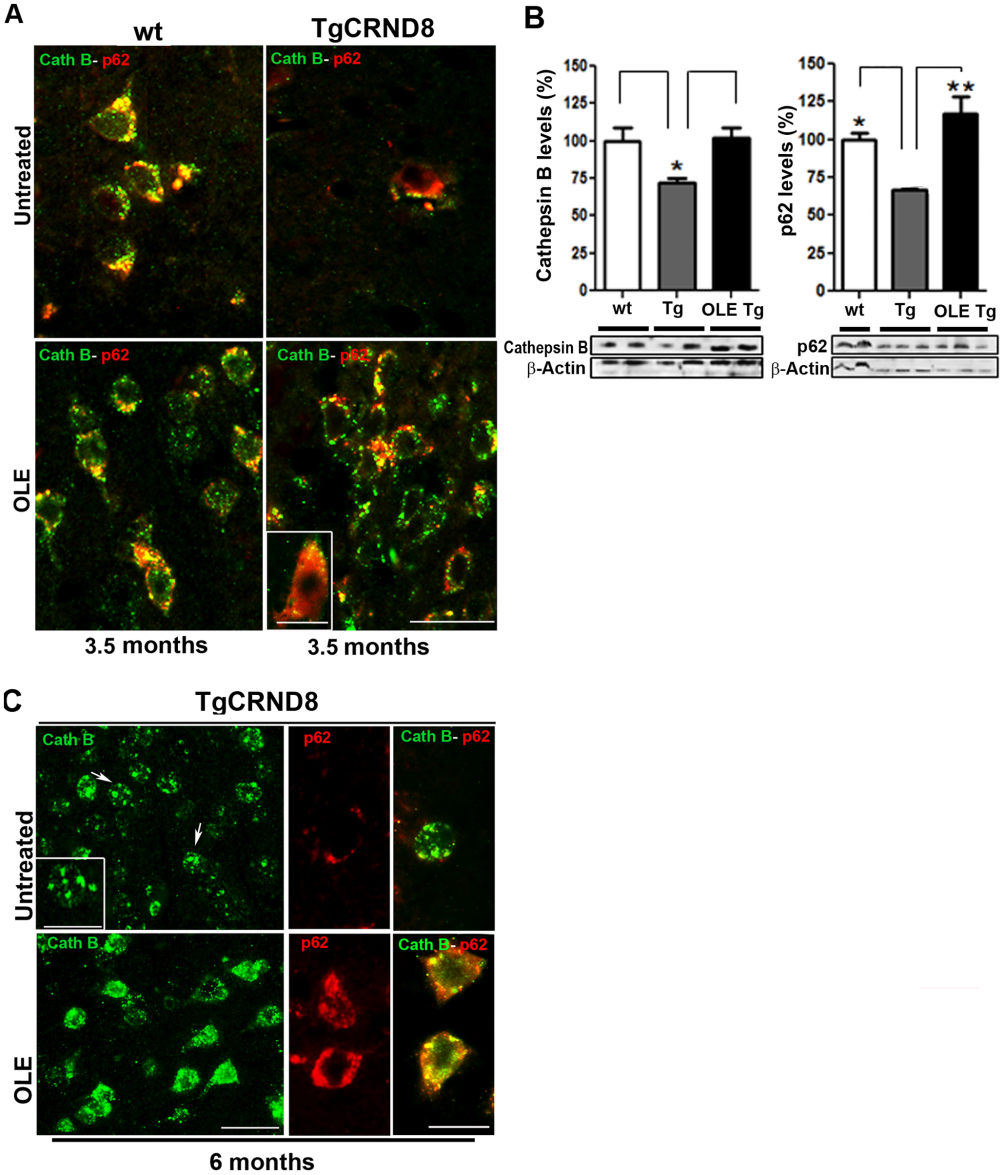
Autophagosome-lysosome fusion in the cortex of untreated and OLE-fed wt and Tg mice. (A) Merge of cathepsin B (green) and p62 (red) immunoreactivity in 3.5-month-old wt and Tg mice. Co-localization between cathepsin B and p62 staining was detected as bright yellow puncta in small-sized lysosomes in the cortex of untreated wt mice and OLE-fed Tg and wt mice (n = 6/group). Scale bar = 25 µm. Inset: high magnification of a p62 and cathepsin B positive neuron. Scale bar = 14 µm. (B) Western blotting analysis of cathepsin B and p62 levels in the cortex of 3.5-month-old wt and Tg mice; wt = pool of untreated and OLE-fed wt mice (n = 10); Tg = untreated and OLE-fed Tg mice (N = 6/group). Both cathepsin B and p62 levels were significantly increased in OLE-fed Tg mice, as compared to untreated Tg mice. Data are representative of four experiments and are normalized for β-actin and reported as mean values ± S.E.M. *P<0.05, ** P<0.01. (C) Single and double fluorescent immunohistochemistry with cathepsin B (green) and p62 (red) Abs in the cortex of untreated and OLE-fed Tg mice. In the untreated Tg mice, bright cathepsin B immunoreactivity occurred in enlarged lysosomal compartments (arrows), p62 immunoreactivity was light and no co-localization between cathepsin B and p62 was found. Inset: high magnification of a cell with cathepsin B-positive giant lysosomes. In the OLE-fed Tg mice, a bright cathepsin B immunoreactivity appeared in small-sized lysosomes, p62 immunoreactivity was greater than in untreated Tg mice and a significant co-localization between cathepsin B and p62 was evident. (n = 6). Scale bars = 25 µm applies for single cathepsin B staining and 10 µm applies to the inset and high magnification images.

To better assess the protective role of the increased autophagy in OLE-fed mice we checked whether it results in autophagosome-lysosome fusion. An intense cathepsin B immunoreactivity was detected as bright puncta in small-sized lysosomes within neurons in the superficial and deep layers of the somatosensory/parietal and entorhinal/piriform cortices of 3.5 month-old Tg mice fed with OLE (Fig. 5A). Significant co-localization was found in these animals by double staining with Abs against cathepsin B and p62, a cargo receptor targeting many cellular substrates for autophagic degradation [44] (Fig. 5A). A similar staining was found in the wt mice treated or untreated with OLE, independently of animal age, as exemplified for 3.5-month-old mice (Fig. 5A), and the quantitative analysis confirmed that cathepsin B reached similar levels in all these mice (Fig. 5B). Altogether, these findings suggest that the autophagosome-lysosome fusion needed for cargo degradation is under way in OLE-fed Tg and wt mice whereas a marked reduction of the cathepsin B expression is present in the same cortical areas of untreated 3.5-month-old Tg mice (Fig. 5B), with no co-localization between cathepsin B and p62, indicating a transgene-associated dysfunction of the autophagic pathway. In the cortex of untreated 6-month-old Tg mice (Fig. 5C), bright cathepsin B immunoreactivity occurred mostly in enlarged lysosomal compartments within neuronal soma (arrows) as already reported for cathepsin D staining in the affected brain regions of 6-month-old Tg mice [6]. As in young Tg mice fed with OLE, also in 6-month-old Tg mice fed with OLE a bright cathepsin B immunoreactivity appeared in small-sized lysosomes, whereas cathepsin B-positive giant lysosomes were almost absent (Fig. 5C). Moreover, in wt mice, either untreated or treated with OLE (Fig. 5A), and in OLE-fed Tg mice of both ages, p62 immunoreactivity was markedly greater than in age-matched untreated Tg mice (Fig. 5A, C), as confirmed by Western blotting of brain extracts, here shown for 3.5-month-old mice (Fig. 5B). A substantial lack of co-localization between cathepsin B and p62 was found in 6-month-old untreated Tg mice (Fig. 5C). As shown for younger mice, also in this case OLE feeding was associated with a recover of p62-cathepsin B co-localization (Fig. 5C, yellow staining), indicating that OLE administration rescues autophagosome-lysosome function also in advanced stages of amyloid deposition.

### OLE Increases Autophagy in N2a Cells

**Figure 6:**
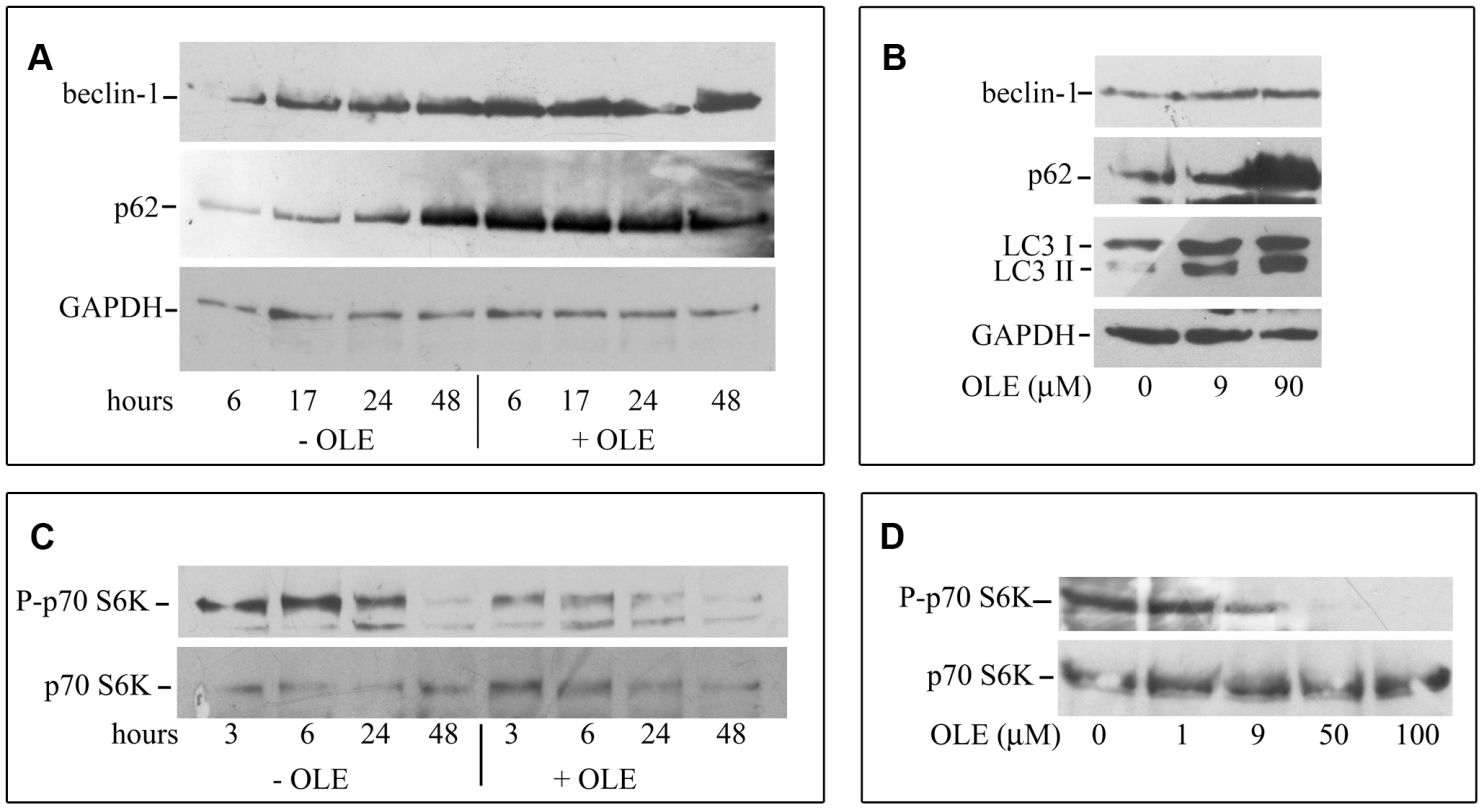
OLE activates autophagy in N2a murine neuroblastoma cells. The cells were exposed to 90 µM (A) or 50 µM (C) OLE for increasing time periods. (B) and (D): the cells were exposed for 6 h to increasing OLE concentrations. Cells were lysed and analyzed by western blotting as described. This is a representative experiment out of three that gave qualitatively identical results.

To confirm that autophagy in mouse brain tissue was really induced directly by OLE, cultured N2a neuroblastoma cells were exposed for various time periods to increasing amounts of OLE. The time-course analysis showed that 48 h cell culturing without medium replacement, resulting in nutrient shortage, was needed to increase the expression of autophagic markers (Fig. 6A). However, cells cultured similarly but exposed to 90 µM OLE displayed increased Beclin 1 and p62 expression as compared to control cells already after 6 h of treatment. The dose-dependence analysis showed that LC3-II/LC3-I ratio and Beclin 1 expression increased already after 6 h of treatment with 9.0 µM OLE; 90 µM OLE administration resulted in a further increase of these markers together with that of p62 (Fig. 6B). Finally, the time- and dose-dependent decrease of phosphorylation of the mTOR substrate p70 S6-kinase in cells exposed for 3–48 h to 50 µM OLE or for 6 h to 9.0–100 µM OLE (Fig. 6C, D) suggests that autophagy activation by OLE proceeds through mTOR inhibition. The Neutral Red (NR) Uptake assay, which measures cell viability in terms of ability to retain the dye inside lysosomes, is particularly appropriate in this context where lysosome integrity is a prominent feature of functional autophagy activation. No significant differences in this assay were found between control and OLE-treated cells exposed for 48 h to 90 µM OLE (OLE = 99.9±0.9110, n = 4, calculated with respect to control taken as 100%). These data further suggest that OLE is directly responsible for autophagy induction, is protective and does not trigger neurodegeneration and cell death as it is the case of several polyphenols [43] and of the prolonged treatment with rapamycin [7].

### OLE Reduces Astrocyte Reaction

**Figure 7:**
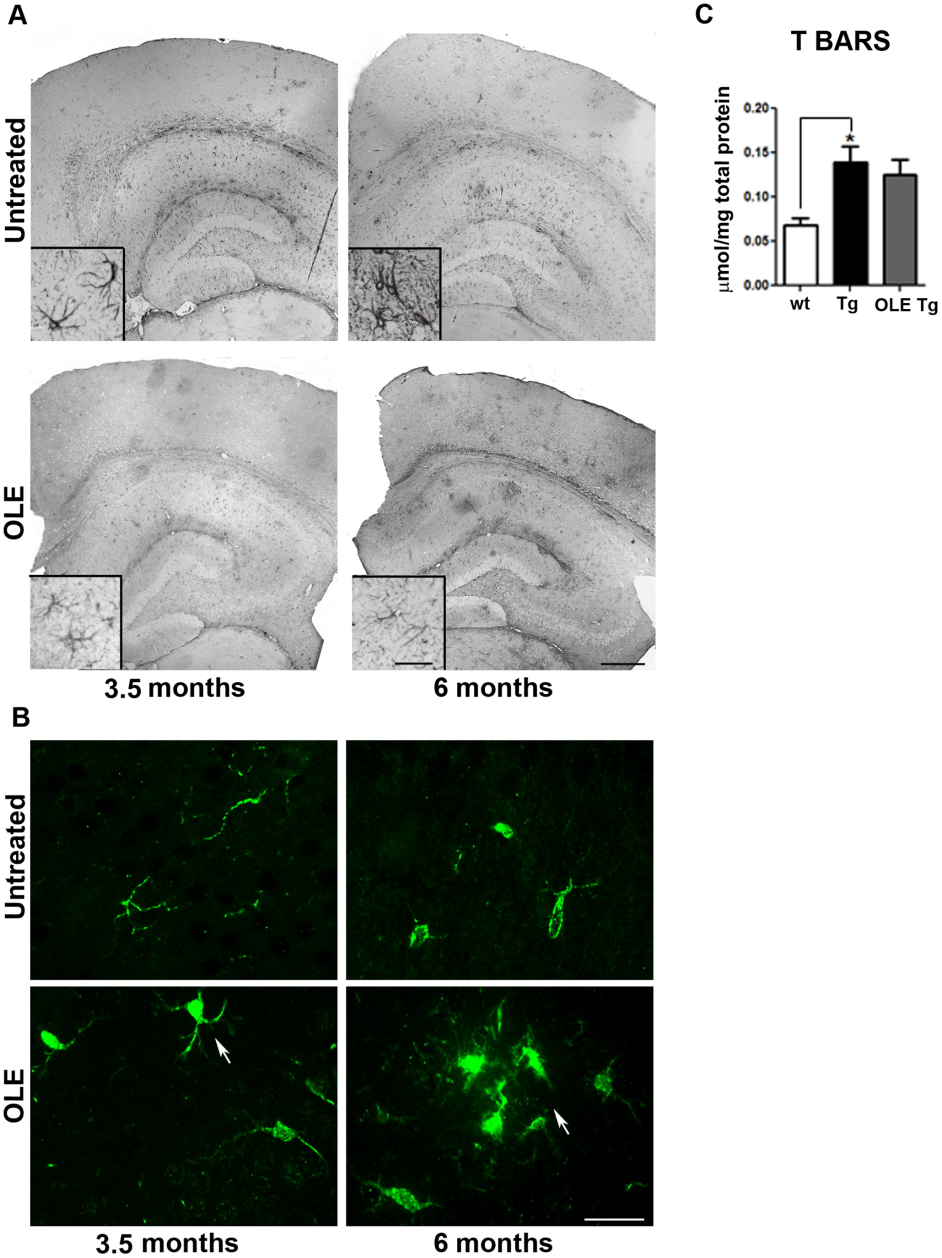
Astrocyte and microglia reaction in the brain of untreated and OLE-fed TgCRND8 mice. (A) reconstruction of representative photomicrographs of GFAP immunoreactivity in the cortex and hippocampus. Hypertrophic astrocytes with long and thick branches were detected in the brain of untreated Tg mice. In the OLE-fed animals the astrocyte reaction was considerably milder. Insets: high magnification of GFAP-positive astrocytes (n = 4–5/group). Scale bars: 500 µm for all reconstructed images and 25 µm for all insets (B) Iba1 immunopositive microglial cells in the cortex. Note the presence of microglia with enlarged cell bodies, thickened and retracted processes (n = 4–5/group) in the cortex of OLE-fed 6-month-old Tg mice. Scale bar: 25 µm applies to all images. (C) TBARS in cortical homogenates of 3.5-month-old mice showing that lipid peroxidation was not significantly reduced by OLE treatment. *P<0.05. Data are reported as mean values ± S.E.M. (n = 3–4 mice/group). Each sample was analyzed in two replicates.

It is widely recognised that amyloid deposits in the brain activate an inflammatory response that contributes to cell sufferance and functional decline [45]. Accordingly, we investigated whether OLE-treatment reduced the inflammatory response in our Tg mice. We detected hypertrophic astrocytes with long and thick branches in the cortex and in all the subregions of the hippocampus of untreated Tg mice (Fig. 7 A). By contrast, in the OLE-fed animals the astrocyte reaction was considerably milder (Fig. 7A), indicating reduction of inflammation. The latter was not the result of the antioxidant power of OLE; in fact, lipid peroxidation in the cortex of 3.5-month-old Tg mice was not significantly reduced by OLE treatment (Fig. 7C). Next, we investigated the effect of OLE administration on microglia morphology. Surprisingly, activated microglia with enlarged cell bodies, thickened and retracted processes or losses of branches were detected in the hippocampus (not shown) and cortex of OLE-fed 6-month-old Tg mice (Fig. 7B). Such a scenario coincided and agreed with the presence of the less dense “fluffy” amyloid plaques described above, suggesting activated microglia involvement in plaque remodelling and phagocytosis. In untreated Tg mice some morphologically activated microglia were detected only in 6-month-old animals while in 3.5-month-old mice resting microglia with thin cell bodies and elongated branches were mostly detected (Fig. 7B). These data support protection by OLE against the inflammatory response to amyloid deposits mainly at the astrocyte level, while microglia activation by OLE can mainly contribute to plaque clearance.

## Discussion

The present study provides acompelling evidence that, in young/middle-aged TgCRND8 mice, diet supplementation for 8 weeks with OLE remarkably improves animal behavior in two memory tests respect to normally fed littermates, with scores reaching those displayed by age-matched wt mice. Improved behavior is accompanied by a significant reduction in Aß40 and Aß42 levels as well as in size and compactness of Aβ plaques and by the presence of fluffy deposits in the older Tg mice. Altogether, our data suggest that OLE treatment reduces *de novo* Aβ deposition and favors preformed plaque disassembly. These results agree with our recently reported findings on a recombinant *C. elegans* strain expressing human Aβ42 in the cytoplasm of muscle cells, showing that worms grown on an OLE-supplemented medium displayed remarkably improved motility and reduced plaque deposition [46].

It has been already reported that the inability to acquire the step down-inhibitory response and to explore a novel object over a familiar one in this mouse model reflects dysfunction of cortical areas [30], [47]; Functional disruption in the neuronal network has repeatedly been reported in AD mouse models [48]. Aberrant neuronal activity and significant reduction of the number of active neurons is particularly present near amyloid plaques, whose presence causes disturbances resulting in abnormalities of whole neuronal networks both in animal models [30], [49] and in asymptomatic patients with amyloid deposits [50]. Our data demonstrating that OLE treatment of young/middle-aged Tg mice results in a concomitant improvement of non-spatial episodic memory and working memory together with amelioration of cortical neuropathological aspects and a remarkable induction of autophagy underscores a tight link between these effects. TgCRND8 mice develop a pattern of Aβ deposition recalling several aspects of human AD. Small size Aß42 immunopositive plaques appear in various brain areas, including the cortex and the hippocampus, by the age of 3 months. As a function of age, they become small-medium to big in size and acquire a compact core, becoming numerous and reaching the maximum roughly by 7–8 months of age [28], [30], [35]. OLE administration with diet to pre-plaque TgCRND8 mice for 8 weeks results in a remarkable reduction of the Aβ load, with a significant decrease of levels, plaque number and area, in agreement with the *in vitro* anti-aggregation effect of OLE we have reported previously [24]. In the OLE-treated older Tg mice, the cortical levels are reduced and the plaques appear less compact displaying ribbon-like and fluffy morphologies in both the cortex and the hippocampus, indicating the occurrence of dual concomitant effects of OLE, prevention of amyloid deposition and disaggregation of preformed plaques. It is increasingly recognised that autophagy protects neurons from Aβ-induced cytotoxicity and that autophagy dysfunction is a molecular link between brain ageing, Aβ accumulation in the brain parenchyma and cognitive impairment [4], [6], [7], [51]. Recently, brain inflammation and Aβ deposition following some triggering insult have been proposed to establish a self-reinforcing cycle integrating the amyloid cascade hypothesis presently considered at the basis of cerebral impairment in AD [52]. Deletion of Beclin 1 in mice has been reported to increase Aβ deposits, to decrease neuronal autophagy and to promote neuronal degeneration, while gene therapy using lentivirus expressing Beclin 1 reduces amyloid pathology in APP transgenic mice [53]. Accordingly, we associate the amelioration of cognitive function of young/middle-aged animals and of neuropathology with a remarkable induction of the autophagic pathway in the cortex of OLE-treated Tg mice. A strong punctuate immunoreactivity and higher levels of autophagosome-lysosome markers, from Beclin-1 to LC3 and cathepsin B, are detected in the cortex and, to a lesser extent, in hippocampal areas of OLE-fed Tg mice at both stages of amyloid deposition, as compared to untreated age-matched Tg mice. In the Tg mice, OLE administration triggers the autophagic machinery to the level detected in control wt mice and leads to the delivery of autophagosome substrates to lysosomes for degradation, as shown by the widespread p62-cathepsin B co-localization. By contrast, in the untreated Tg mice autophagosome-lysosome markers analysis reveals a clear dysfunction of the autophagic pathway. The cortical and hippocampal astrocyte reaction detected in untreated Tg mice is strongly ameliorated by OLE administration. This anti-inflammatory activity of OLE treatment is not associated with the known OLE anti-oxidant activity, which plays a minor role, if any, in all the effects we have found. In fact, the OLE-induced migration of microglia to the amyloid plaques favoring phagocytosis of amyloid deposits could maintain/produce an oxidant environment thus underlying the absence of significant anti-oxidant effects of OLE administration. In addition, since OLE is highly prone to oxidation, it probably loses its anti-oxidant power before reaching mice brain.

Our data strongly support the idea that OLE treatment combats Aβ neurotoxicity and Aβ-induced cognitive impairment in our mouse model through reduction of plaque load and consistency resulting from a strong induction of autophagy, concomitantly with a recovery of the lysosomal system (whose dysfunction is one of the earliest disturbances that occur in AD [54] and from microglia activation. The two responses could co-operate to the final protective effect; in fact, the migration of the activated microglia to the Aβ deposits would result in plaque disassembly and fragmentation; the activated autophagy would complete the protective response explaining reduced plaque burden, dimensions and compactness The beneficial effects of the MD on attenuating cognitive impairment, AD-like pathology and neurodegenerative diseases has been repeatedly reported [13]–[18]. Overall, the behavioral and histological data presented in our study support the notion that the beneficial effects of the MD can, at least in part, be traced back to the intake of EVOO and its main polyphenol, OLE. We also provide a molecular explanation of the protective effects elicited by OLE against age-related and AD-type neurodegeneration. Our findings are strengthened by previous data indicating that in rat and humans, orally administered olive oil phenols, including OLE, its glycoside and/or one of its derivatives arising from tissue metabolism, are intestinally absorbed skipping degradation by microorganisms in the colon, cross the blood-brain barrier and are found inside brain parenchyma [55], [56]. In conclusion, our results support the possibility that dietary supplementation of OLE may prevent or delay the occurrence of AD and may reduce the severity of its symptoms.
